# Extended One-Way Waveguide States of Large-Area Propagation in Gyromagnetic Photonic Crystals

**DOI:** 10.3390/nano14221790

**Published:** 2024-11-07

**Authors:** Xiaobin Li, Chao Yan, Zhi-Yuan Li, Wenyao Liang

**Affiliations:** School of Physics and Optoelectronics, South China University of Technology, Guangzhou 510640, Chinaphzyli@scut.edu.cn (Z.-Y.L.)

**Keywords:** extended one-way waveguide states, gyromagnetic photonic crystals, large-area propagating property, topological high-throughput transmission

## Abstract

We propose extended one-way waveguide states of large-area propagation in a photonic crystal waveguide consisting of two honeycomb gyromagnetic photonic crystals with opposite external magnetic fields. When the width of the waveguide is small enough, the edge states along both boundaries of the waveguide couple with each other strongly and thus create the so-called extended one-way waveguide states. Of note, this structure supports both even and odd extended states, which can be excited under different excitation conditions. For the odd mode, electromagnetic waves have opposite phase distributions along the centerline of the waveguide on both sides, while for the even mode, they have in-phase distributions on both sides. In addition, the odd and the even modes both have the large-area propagating property. Moreover, we have carried out a microwave experiment to verify the simulation results. The measured transmission spectrum shows that the structure has strong non-reciprocity, and the measured electric field distributions of the even and odd modes prove that it supports excellent large-area transmission behaviors. These results provide feasible ideas for achieving topological high-throughput transmission.

## 1. Introduction

Photonic crystals (PCs), as a typical micro-/nano-artificial structure [1,2], possess rich design freedom and have unique advantages in the realization of topological photonic devices. In recent years, by drawing analogies from the band theory and topological states in electronic systems, researchers have proposed topological PC structures analogous to the quantum Hall effect, quantum spin Hall effect, and quantum valley Hall effect. Correspondingly, they have achieved topologically protected chiral unidirectional edge states [3,4], pseudo-spin edge states [5], and valley edge states [6,7,8]. In 2008, Haldane and Raghu from Princeton University first proposed that in a Gyromagnetic PC (GPC) composed of Faraday effect materials, applying an external magnetic field (EMF) will break its time-reversal symmetry [3], and thus generate chiral unidirectional edge states similar to the electronic quantum Hall effect. Its topological properties can be characterized by a non-zero Chern number [9] of the band structure. By using these edge states, various high-performance topological photonic devices such as topological laser [10,11,12], unidirectional beam splitter [13,14], and topological add drop filter [15,16,17] have been proposed. In 2020, antichiral edge states were theoretical proposed [18] and experimentally verified [19], which also have strong robustness against various defects.

The chiral and antichiral topological edge states provide unidirectional, backscattering immune energy transport at the strip edge. However, from the previous studies [18,19,20,21,22], these chiral and antichiral topological edge states rely solely on the boundary regions of the structure for electromagnetic wave transmission, which results in low transmission flux and poor space utilization. To improve the space utilization, researchers have begun to investigate mechanisms for large-area unidirectional transmission [23,24,25]. In 2021, C. T. Chan et al. proposed a sandwich waveguide structure composed of two magneto-optical PCs and one PC with Dirac dispersion. This structure enabled unidirectional transmission with extended boundary states to support a large mode field distribution [23]. Later, Y. Yang et al. introduced valley PCs on either side of the Dirac dispersion PC, which change the valley boundary state into an expanded mode field [26]. Recently, our group proposed unidirectional transmission of waveguide states with a large mode field and pseudo-spin-momentum locking by integrating Dirac dispersion PCs into pseudo-spin magneto-optical PCs [27]. Although these works explored new ways to expand the transmission area of the waveguide, they all used a sandwich structure, which led to complex analysis and fabrication. Differently, here, we proposed a new mechanism for large-area propagation by generating extended one-way waveguide states by using line defect PC waveguide. The structure has only two honeycomb GPCs, which is much simpler and easier for fabrication than those sandwich structures.

In this work, we propose extended one-way waveguide states which are realized in a waveguide structure composed of two honeycomb GPCs with opposite EMFs. When the waveguide width is small enough, there exist both even and odd extended states. They can be excited to support large-area one-way propagating by sources with in-phase and opposite phases, respectively. Moreover, we design an experiment to explore the large-area propagating behaviors. The transmission spectrum and the eigenmodal fields of the experiment agree well with the simulation results. These results provide a feasible idea for reaching topological high-throughput transmission.

## 2. Materials and Methods

### 2.1. Basic GPC Structure

**Figure 1:**
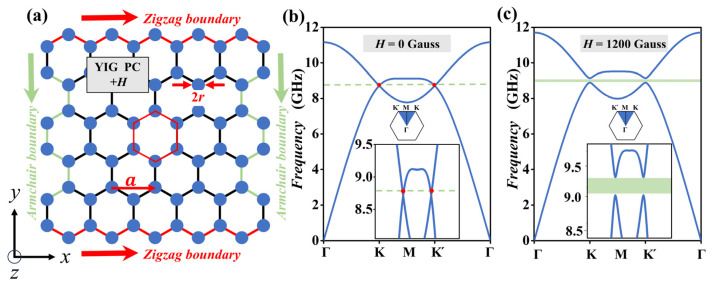
GPC structure and band structure. (**a**) The honeycomb GPC structure with the zigzag edges along the x direction. The unit cell (marked by red regular hexagon) is used to calculate the bulk band structures shown in (**b**,**c**). (**b**) The bulk band structure of TM mode without an EMF. The red dots indicate the two Dirac points which are connected by a green dotted line. (**c**) The bulk band structure of the TM mode with an EMF of *H*_0_ = +1200 Gauss. The green rectangle represents the band gap. The insets are the larger image of the band structure.

We start with a 2D honeycomb GPC which is composed of cylindrical yttrium iron garnet (YIG) rods immersed in air. As shown in Figure 1a, the relative permittivity and the radius of the YIG rods are *ε* = 14.5 and *r* = 0.15*a*, where *a* = 10 mm is the lattice constant of the GPC. The honeycomb GPC has two typical boundary configurations. One is the zigzag boundary along the *x* direction, while the other one is the armchair boundary along the *y* direction, as shown by the red and green arrows in Figure 1a, respectively. When no EMF is applied, the magnetic permeability of YIG rods is *μ* =1. However, when an EMF of *H*_0_ = +1200 Gauss is applied to the YIG rods along the +*z* direction, the gyromagnetic anisotropy is strongly induced to break time-reversal symmetry and the permeability becomes a tensor as follows [28,29]:

(1) $$\hat{\mu }=\left(-\begin{array}{ccc} \mu_{r} & j\mu_{k} & 0\\ ju_{k} & \mu_{r} & 0\\ 0 & 0 & 1 \end{array}\right)$$

where *μ_r_* = 1 + *ω_m_*(*ω*_0_ + *jαω*)/[(*ω*_0_ + *jαω*)^2^ − *ω*^2^)] and *μ_k_* = *ωω_m_*/[(*ω_0_* + *jαω*)^2^ − *ω*^2^)], *γ* = 2.8 × 10^6^ Hz/Oe is the gyromagnetic ratio, *ω*_0_ = 2π*f*_0_ = 2π*γH*_0_ is the resonance frequency, *ω_m_* = 2π*f_m_* = 2π*γM*_0_ is the characteristic circular frequency, with *M*_0_ = 1750 Gauss being the saturation magnetization, and the damping coefficient is *α* = 3 × 10^−4^. The bulk band structures of transverse magnetic (TM) mode with *H* = 0 Gauss and +1200 Gauss are calculated by the honeycomb unit cell (marked as a red hexagon in Figure 1a) by the finite-element method, as shown in Figure 1b,c, respectively. In addition, all six boundaries of the unit cell are set into three pairs of parallel Floquet periodic boundaries.

Figure 1b is the bulk band structure of GPC without an EMF. Obviously, the band gap will not exist and in the first Brillouin zone, two bands couple with each other at two Dirac points of *K* and *K’*, which is marked as the green line intersecting with the band of two red dots. The larger image of Figure 1b shows that the frequencies of two Dirac points are 8.85 GHz. Figure 1c shows the bulk band structure of GPC, which is applied with *H*_0_ = +1200 Gauss. Two coupled points at Dirac points *K* and *K’* are broken and the two bands are separated from each other (marked as the green zone). The larger image of Figure 1c shows that the opened band gap appears from 9.02 to 9.28 GHz with a bandwidth of 0.26 GHz. 

### 2.2. Projected Band Structure and Physical Mechanism Analysis 

The Chern number *C_n_* and the gap Chern number *C_gap_* can describe the topological properties of the structure [28,30]. The above honeycomb GPC is applied with *H*_0_ = +1200 Gauss in Figure 1c. The *C_n_* of the first and second bands are −1 and +1, respectively [31]. The sign of Δ*C_gap_* determines its propagation direction of the waveguide and the value of |Δ*C_gap_*| determines the number of one-way edge states. 

**Figure 2:**
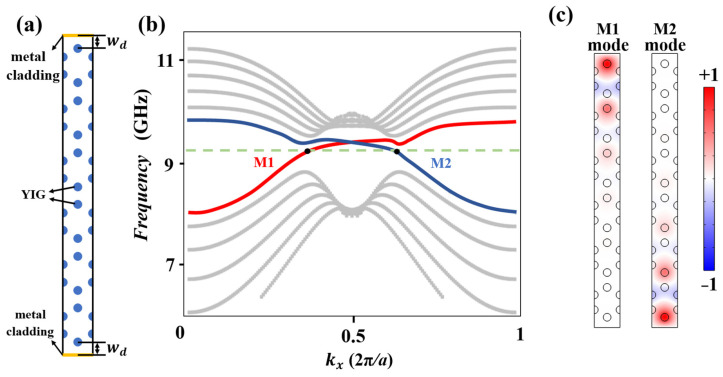
GPC supercell structure, band structure and eigenmodal fields. (**a**) The supercell of the honeycomb GPC structure which is used to calculate the projected band structure. Solid yellow lines at both sides indicate the metal claddings. (**b**) The projected band structure of TM mode with an EMF *H*_0_ = +1200 Gauss. A green dotted line represents a typical frequency of *f_s_* = 9.2 GHz, which intersects with two bands at M1 and M2 points, respectively. (**c**) The eigenmodal fields of M1 and M2 modes.

Firstly, we study the characteristics of the projected band structure. To further explore the topological property, two metallic claddings (denoted by yellow color) are placed close to the zigzag edges of the 2D honeycomb GPC to form two isolated unilateral waveguides with a width of *w_d_* = *a*. Figure 2a shows the structure of supercell. It is remarkable that the upper and lower boundaries of the supercell are perfect electric conductors (PECs) which are used to avoid the energy leaking into the air. The left and right boundaries are Floquet periodic boundaries. Figure 2b shows the projected band structure of the upper and lower waveguides with Δ*C_gap_* of −1 and +1 (marked by red and blue line, respectively). There exists a topological band gap from 8.87 GHz to 9.50 GHz. The red and blue curves in the band gap represent two one-way edge states with counter-propagating directions. To demonstrate the characteristics of these waveguide modes in depth, we choose the central frequency (*f_s_* = 9.2 GHz) which intersects with the red and blue dispersion curves at points M1 and M2 for further discussions. Figure 2c shows the eigenmodal field of |*E*| of the M1 and M2 modes, respectively. Obviously, the eigenmodal fields are localized at the edges’ region and gradually decrease towards the center of the supercell.

**Figure 3:**
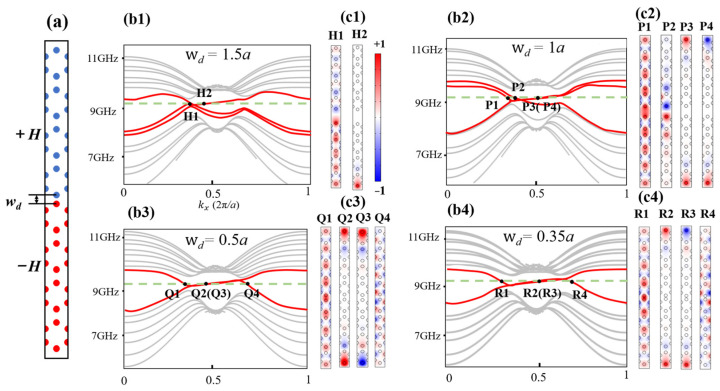
Schematic illustration, band structure and eigenmodal fields of the supercell. (**a**) Schematic illustration of the supercell. +*H* and −*H* are applied to the upper and lower cells (mark by blue and red, respectively). (**b1**–**b4**) TM band structure for *w_d_* = 1.5*a*, 1*a*, 0.5*a* and 0.35*a*, respectively. (**c1**–**c4**) Eigenmodal fields at 9.2 GHz with different *w_d_*.

Here, we discuss the physical mechanism of the extended one-way waveguide states. As shown in Figure 3a, we construct a supercell by the upper and lower PCs with opposite EMFs of +*H* and −*H*, respectively (mark by blue and red, respectively). The distance between the nearest red and blue cylinders is *w_d_*. As *w_d_* decreases from 1.5*a* to 0.35*a*, the band structures show evident differences, as shown in Figure 3(b1–b4). We take *f_s_* = 9.2 GHz for detailed analyses. When *w_d_* = 1.5*a* [Figure 3(c1)], the width of the waveguide is large enough so that the interaction between the upper and lower PCs is negligible. There only exists one edge state H2 and one transmission state H1. When *w_d_* decreases to 1*a*, the interaction effect between the upper and lower PCs appears and results in an even P1 mode and odd P2 mode, as well as a couple of degenerate antichiral edge states [i.e., P3(P4)] which co-propagate at the upper and lower boundaries of the structure. However, the odd P2 mode still mainly localizes at the center of the supercell, as shown in Figure 3(c2), meaning the interaction is not strong enough. Then, we continue to decrease the *w_d_* to observe the evolution of the eigenmodal field. Interestingly, when *w_d_* ≤ 0.5*a*, the width of the waveguide is small enough to cause a strong interaction; so, the odd mode and even mode both become the extended one-way waveguide states (Q1, Q4 for *w_d_* = 0.5*a*; R1, R4 for *w_d_* = 0.35*a*), as shown in Figure 3(c3,c4). In addition, the degenerate antichiral edge states (Q2, Q3) and (R2, R3) still exist. It is emphasized that for these extended waveguide states, their energy is no longer localized at the center of the supercell but spread throughout the entire structure. This property holds great potential in constructing a transmission structure with one-way large-area propagation, which is beneficial for enhancing spatial utilization. For simplification, we will choose the waveguide structure with *w_d_* = 0.35*a* for further discussions.

### 2.3. Design of Experiment Scheme

**Figure 4:**
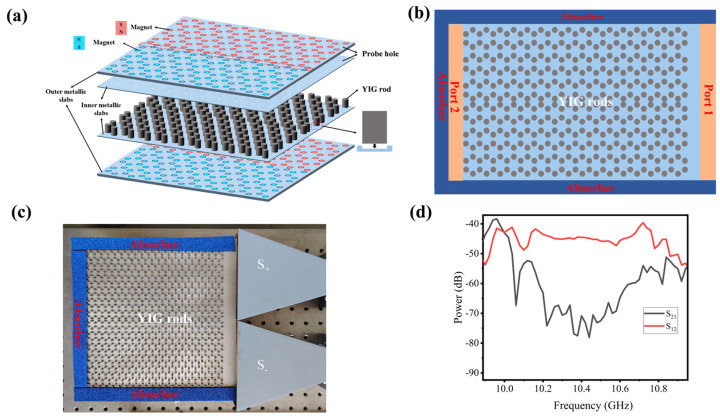
(**a**) Three-dimensional schematic diagram of the realistic experimental model. The red and light-blue colors indicate different combinations of magnetic poles *N* and *S*. The white dots on the upper two metallic slabs represent the holes for probing the EM waves in the GPC structure. (**b**) Horizontal schematic diagram of the realistic experimental model. (**c**) The fabricated sample. (**d**) Transmission spectrum of the experiment structure.

We theoretically analyzed the implementation of the extended one-way large-area waveguide states of honeycomb GPCs in detail. Here, we come up with a realistic experimental design to verify the simulation results. As shown in Figure 4a, the inner pair of metallic slabs are set in the upper and lower parts of the GPC structure to impede the electromagnetic waves from transporting in the *z* direction, while the outer pair of metallic slabs are carved out as honeycomb arrays to locate the magnet arrays, and then cover from the top and the bottom of the inner structure to complete sample fabrication. The positive and negative EMFs are produced by the different combinations of the magnetic poles of magnets (i.e., SN or NS combinations). Figure 4b shows the horizontal schematic diagram of the realistic experimental model for verifying the extended waveguide states. Three absorbers are set around the GPC structure to prevent the leakage of electromagnetic waves. The input port 1 and output port 2 (colored in the orange rectangles) are arranged on the right and left sides of the GPC structure. Figure 4c is the fabricated sample. We use vector network analyzer (Keysight P9374A, Santa Rosa, CA, USA) to observe the transmission of the signal. Two horn antennas (JUNCOAX-LB39591517, Shanghai, China) are used as two plane wave sources (marked with S+ and S−) with the same intensity but opposite phases at port 1 to excite the odd R4 mode. The transmission spectrum is measured and shown in Figure 4d. The topological band gap of the experiment appears from about 10.18 GHz to 10.66 GHz, meaning that the experimental band gap is slightly narrower than that in the simulation. This phenomenon is also affected by the uneven magnetization and the inaccurate geometric parameters of the YIG rods during fabrication. The red curve represents the energy ratio from port 1 to port 2, while the black curve is that from port 2 to port 1. They are marked as S_12_ and S_21_, respectively. The difference between the leftward and rightward transmissions between 10.18 and 10.66 GHz is 35 dB or so, exhibiting strong nonreciprocity, which can well satisfy practical needs. It should be noted that the topological band gap shifted about 1 GHz towards higher frequency since the magnetic field in the actual experiment is larger than that in the simulations.

## 3. Results

### 3.1. Numerical Simulations of the One-Way Large-Area Waveguide Extended States

**Figure 5:**
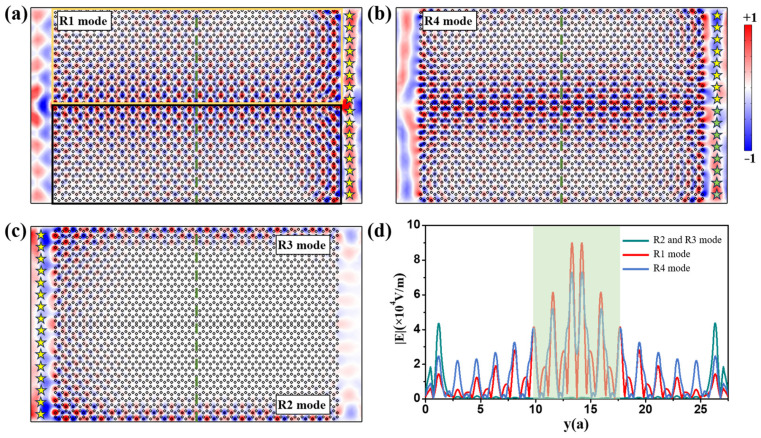
*E*_z_ fields and the amplitude of electric fields (|*E*|). (**a**–**c**) The intensity distributions of *E_z_* fields of R1, R4, R2 and R3 modes, respectively. Stars of different color show the line sources are in opposite phases. (**d**) The amplitude of electric fields (|*E*|) along the green dotted lines of R1, R4, R2 and R3 modes, respectively. The green rectangle represents the half-peak range of electric fields.

In order to further explore the transmission behaviors of the extended one-way waveguide states of R1 and R4 modes, we construct a waveguide consisting of two identical GPCs with *H*_0_ = +1200 and −1200 Gauss as denoted by the yellow and black frames in Figure 5a, respectively. The size of the waveguide is 40*a* × 16$\sqrt{3}$*a*. A series of 2D line sources (marked by stars) oscillating at *f_s_* = 9.2 GHz are placed at equal spacing on the right side of the structure, and they are longitudinally symmetrical regarding the waveguide, as shown in Figure 5a,b. The even R1 mode is excited when all line sources are in-phase. Conversely, the odd R4 mode is excited when the line sources on the upper and lower parts are in opposite phases. Figure 5a,b clearly shows that the R1 and R4 mode can propagate leftwards over a large area of the GPC rather than being localized within the waveguide, which is consistent with the eigenmodal field results in Figure 3(c3,c4). Being completely different from Figure 5a,b, when the line sources are placed on the left side of the GPC, the antichiral edge states of R2 and R3 are excited on the upper and lower boundaries of the GPC, respectively, and they co-propagate rightwards. These simulation results clearly demonstrate the different one-way transmission behaviors of these four waveguide modes [i.e., extended one-way waveguide states (R1, R4) and antichiral states (R2, R3)]. Figure 5d shows the amplitudes of electric field (|*E*|) of these four waveguide modes along the dotted green lines at *x* = 13.5*a* in Figure 5a–c. On the one hand, the |*E*| peaks of the R1 and R4 modes (colored in red and blue, respectively) are distributed near the waveguide with values of 9 × 10^4^ V/m and 7.5 × 10^4^ V/m, respectively. On the other hand, the |*E*| decreases slowly on both sides, showing the characteristics of Gaussian distribution. We can roughly estimate that the half-peak range is from *y* = 9.8*a* to 17.6*a* (marked by the green rectangle) with a width of 7.8*a*. In addition, it is worth noting that there exist two small peaks in the |*E*| curves of the R1 and R4 modes and their positions are consistent with the maximum peaks of the R2 and R3 modes (colored in dark green), indicating that the energy of the R1 and R4 modes diffuses to the upper and lower boundaries and slightly excites the antichiral R2 and R3 modes. It should be noted that the coupling strength between the even mode and antichiral modes is weaker than that between the odd mode and antichiral modes because the even mode decays faster than the odd mode along the y direction, as shown in Figure 5d.

### 3.2. Experimental Results of the One-Way Large-Area Waveguide Extended States

**Figure 6:**
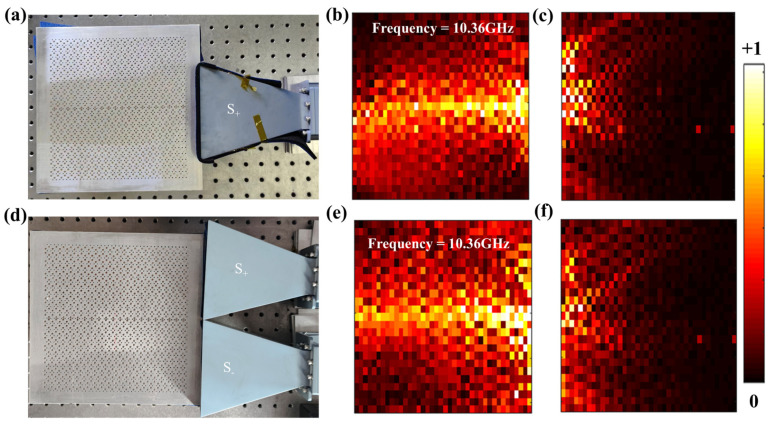
Experimental setups and measured results. (**a**) Schematic diagram for the even R1 mode excited by S+. (**b**,**c**) The measured field distributions of the R1 mode at 10.36 GHz when the source is placed on the right and left side, respectively. (**d**) Schematic diagram for the odd R4 mode excited by S+ and S− with same intensity but opposite phases. (**e**,**f**) The measured field distributions of the R4 mode at 10.36 GHz when the sources are placed on the right and left side, respectively.

Next, we carry out experiments to demonstrate the field distributions of both the even and odd extended waveguide states. The MATLAB program was used for data processing. The experiment setups and measured results are shown in Figure 6. The even and odd modes are excited by one plane wave source (S+) or two plane wave sources with opposite phases (S+ and S−), respectively (Figure 6a,c). In Figure 6b,e, we measure the field distributions of the R1 and R4 modes at 10.36 GHz by using the point-by-point scanning method. The experimental results demonstrate that they can both large-area propagate leftwards. Conversely, when the sources are placed on the left side of the structure, the measured results in Figure 6c,f clearly show that no electromagnetic wave can propagate rightwards. These experimental results prove that the structure supports excellent one-way large-area propagation of electromagnetic waves, which agrees well with the previous simulation results.

## 4. Conclusions

In conclusion, we realized the one-way large-area extended states in a GPC waveguide. When the width of the waveguide is small enough, the interaction between the upper and lower GPCs can create even and odd extended waveguide states simultaneously. They both support large-area one-way transmission within the topological band gap. Moreover, we demonstrated the large-area propagation phenomena of the even and odd states by microwave experiments. The simulated and the experimental results both prove the excellent large-area transmission performance of the proposed structure. Our work has improved the spatial utilization of the waveguide structure and paves the way for creating large-area one-way states. These excellent properties of such extended waveguide states support high-throughput transmission and the design of high-performance topological optical devices such as a Y-shaped splitter and a Z-shaped router. Moreover, the idea of extended one-way waveguide states can also be further extended to the visible or infrared light bands by reducing the lattice constant to the micro/nano scales with suitable materials.

## Data Availability

The data presented in this study are available on request from the corresponding author.
